# Nordic walking with an integrated resistance shock absorber affects the femur strength and muscles torques in postmenopausal women

**DOI:** 10.1038/s41598-022-24131-7

**Published:** 2022-11-22

**Authors:** Krystian Wochna, Małgorzata Ogurkowska, Piotr Leszczyński, Rafał Stemplewski, Anna Huta-Osiecka, Anna Błaszczyk, Jacek Mączyński, Alicja Nowak

**Affiliations:** 1 Laboratory of Swimming and Water Lifesaving, Poznan University of Physical Education, Krolowej Jadwigi 27/39, 61-871 Poznan, Poland; 2 Department of Biomechanics, Poznan University of Physical Education, 61-871 Poznan, Poland; 3 Department of Rheumatology, Rehabilitation and Internal Medicine, Poznan University of Medical Sciences, 61-701 Poznan, Poland; 4 Department of Digital Technologies in Physical Activity, Poznan University of Physical Education, 61-871 Poznan, Poland; 5 Department of Hygiene, Poznan University of Physical Education, 61-871 Poznan, Poland

**Keywords:** Medical research, Rheumatology, Risk factors

## Abstract

Deterioration of the structure and function of the musculoskeletal system represents a significant problem during aging and intervention with a suitable load of physical activity may improve the quality of life. Nordic walking (NW) has become a popular and easily accessible form of activity, especially for older adults people around the world. Thus, the purpose of the study was to evaluate the influence of an Nordic walking training program with classic poles (NW) and with integrated resistance shock absorber (RSA) on bone mineral density and the peak torques of upper limb muscles and to compare the effects of both intervention programs. 25 women were randomly assigned to two training groups: 10 subjects using RSA (68 ± 4.19 years) and 15 subjects using NW poles (65 ± 3.40 years), which completed 8 weeks of training program. The hip, spine and forearm areal bone mineral density, torques of the flexors and extensors at the elbow and shoulder joints were measured before starting the training programs and after their completion. The most significant effect was found in differences between the two groups of women with respect to the femur strength index (p = 0.047) and the ratio of the flexors to extensors in the elbow (p = 0.049) and shoulder (p = 0.001) joints and peak torque of flexors in the shoulder joint (p = 0.001) for the left arm. A significant difference was also found in the index of torque asymmetry of flexors in the shoulder joint (p = 0.002). The study shows that Nordic walking with RSA poles for postmenopausal women led to beneficial changes in the femur strength index. However, we found no significant influence on bone mineral density values measured on the whole body, the femoral neck, forearm or lumbar spine regions. The occurrence of asymmetry in biomechanical muscle parameters, which was observed using RSA poles, may suggest the necessity of systematic controlling the gait technique to avoid the adverse consequences of asymmetrical rotation of the lumbar spine.

## Introduction

Physical activity as prophylaxis of sarcopenia and osteoporosis is a very important and valuable part of the life of older adult women^1^. However, not every form of physical activity may be appropriate, and some may put too much strain on the joints and bones, especially for people with a low fitness level or who are already experiencing negative changes in their musculoskeletal system^2^.

Nordic walking (NW) has become a popular form of activity that could be integrated into the daily life of the older adult population and in physical rehabilitation^3,4^. It is assumed that NW poles provide additional mechanical stimuli and involve muscles which are not used during normal walking^5–7^. Previous studies have shown the beneficial effects of NW programs on biomechanical^3^ and physiological^4^ parameters. It has been observed that NW training increased muscle activity in the lower extremities compared with walking, particularly on level ground^8^. In their meta-analysis, Rodrigues et al.^9^ evaluated the risk of fractures and confirmed that NW may improve physical functioning and BMD in the lumbar spine, but they suggested that more evidence is needed. On the other hand, Pellegrini et al.^10^ indicated that modifying the NW technique (weak poling action, straight-upper limbs moving the shoulders, elbow flexion–extension pattern, shoulder freeze) elicits lower muscular activation and metabolic responses to the basic NW technique. Park et al.^11^ indicated that vertical ground reaction force is a very important part of NW training. Kato et al.^12^ proved that the ground reaction forces transmitted through the poles to the radius stimulate bone formation, particularly in the ultra-distal radius in young women.

One of the interesting forms of NW development is walking using poles with an integrated resistance shock absorber (RSA). RSA is a built-in elastic tape between two permanent elements inside the poles, which allows additional resistance to be obtained and it increase the overall intensity of exercise. Marciniak et al.^13^ suggested that RSA provides additional resistance during marching, which causes increased muscle activation and results in improved muscle strength. Resistance exercises are recommended to alleviate osteosarcopenia^14^. There are a few studies regarding the effects of exercise with an integrated RSA^13,15,16^. However, we did not find any study including measurement of BMD or biomechanical changes in this kind of training, which appears to be important and interesting. It can be assumed that training with RSA poles, with additional resistance, will cause a significant response of the musculoskeletal system. Therefore, the purpose of the present study was to evaluate and to compare the influence of NW training with classic poles and those with integrated RSA on bone mass and the peak torques of upper limb muscles.

## Methods

### Participants

Subjects were recruited by advertisements in local media and at information events.

Women aged 60 years and older were included in the study. Initially, 40 women who declared good health status without inflammatory disorders based on medical consultation were qualified to participate in the project. A medical history and cardiological examination were used to qualify participants for the training program. The study included women declaring no musculoskeletal diseases that prevent independent movement, no giant obesity, cancer disease, and unstable ischemic heart disease. All subjects declared that they did not have a history of participating in professional sports. The subjects were asked not to change their dietary habits for the duration of the project and not to perform any additional physical activity, except for the one carried out in the research project.

Taking into account one-factor effect of time in repeated measures (lack of data connected to effect size for two-factors study structure “group” × ”time” in case of two different intervention), range of number of subjects is estimated from 13 to 22 with power statistics = 0.8 and alpha level = 0.05. Women were randomly assigned to two groups with a different training program—walking with classic poles (NW) and with RSA poles. Randomization was performed as a simple random allocation; each subject identifier was forwarded to a person who was not involved in conducting the study, and who performed the randomization blindly using a computer list.

Participants who failed to abide by the research protocol and declared that during the study period they had systematically participated in other physical activity classes (n = 10) or had low attendance (less than 80%) at the training sessions (n = 2) or did not appear on the second term of research (n = 3) were excluded from project. Moreover, for one person, it was impossible to collect complete results for anatomical reasons. Four women who declared taking medication drugs for osteoporosis were enrolled because during the analysis of the research data, it was noticed that these drugs do not cause significant changes in the test results. Finally, 25 women (10 RSA; 15 NW) aged 61–75 were subjected to statistical analyses. An information sheet was provided to each woman approached to participate in the study and on their agreeing to participate, informed written consent was obtained. Moreover, informed consent from study participants for publication of identifying images in an online open-access publication was obtained. The entire study was conducted from the 13th of February 2019 until the 17th of April 2019. All methods were performed in accordance with the relevant guidelines and regulations.

Before and after the training intervention, somatic features, bone mass and biomechanical parameters were assessed.

### Training program

The research training program^16^ lasted 8 weeks (16 training sessions, twice a week). Both groups of women, using classic NW and RSA poles, participated in training sessions at the same time. Women from the RSA group used poles with an integrated RSA with an elastic resistance of 4 kg (Slimline Bungy Pump, Sports Progress International AB, Sweden). The technique of marching with RSA poles has been applied according to the description by Marciniak et al.^13^. At the beginning of each training session, for 10–15 min, the women performed warm-up exercises (vigorous body movements). Then, after each half of the distance the participants stopped marching and for 15 min performed strength exercises and balance training. Strength exercises were based on overcoming the elastic resistance of the shock absorber of the RSA poles in various positions of the body (8 to 12 repetitions). The exercises focused on the work of the muscles of the upper limbs and the trunk. After completing the marching distance, static stretching exercises were undertaken. During the training program, the walking distance gradually increased, from 3.5 to 4.5 km (speed of about 6 km/h). During the training program exercise intensity increased from 50 to 70% HRR, measured using a heart rate monitor (Polar Electro Oy, Kernpele, Finland)^15^. The minimum attendance was assumed at the level of 80% (13 training sessions). The trainer had the appropriate qualifications (International Nordic Walking Association). The participants declared that they did not have previous experience of the NW technique. Before the training program, during a 60-min session, women were trained in the correct marching techniques.

### Bone density measurements

The DXA scans were held on one day, a few days before the training intervention and after their completion. Measurements were performed as previously described in Wochna et al.^17^. Areal bone mineral density (aBMD) was measured using dual X-ray absorptiometry (DXA) on the whole body, the left hip (total hip and neck), the lumbar spine (L_1_–L_4_) and forearm, as well as total fat and lean body mass were determined. The DXA measurements were acquired using a Lunar Prodigy Advance densitometer (General Electric, USA). The DXA measurements were expressed as areal bone mineral density (aBMD, g/cm^2^) and T-score. All scans were taken by the same technician, using the same device, which was calibrated daily. Quality control of the DXA scanner was performed in accordance with the manufacturer’s instructions, and scan analyses were performed using integrated software, according to the manufacturer’s recommendations. The femur strength index of the left hip was automatically calculated using the ratio of estimated compressive yield strength of the femoral neck to the expected compressive stress of a fall on the greater trochanter^18^. This algorithm considers the shape of the proximal femur as well as the cross-sectional moment of inertia in the estimate^19^.

### Biomechanical measurement procedures

**Figure 1:**
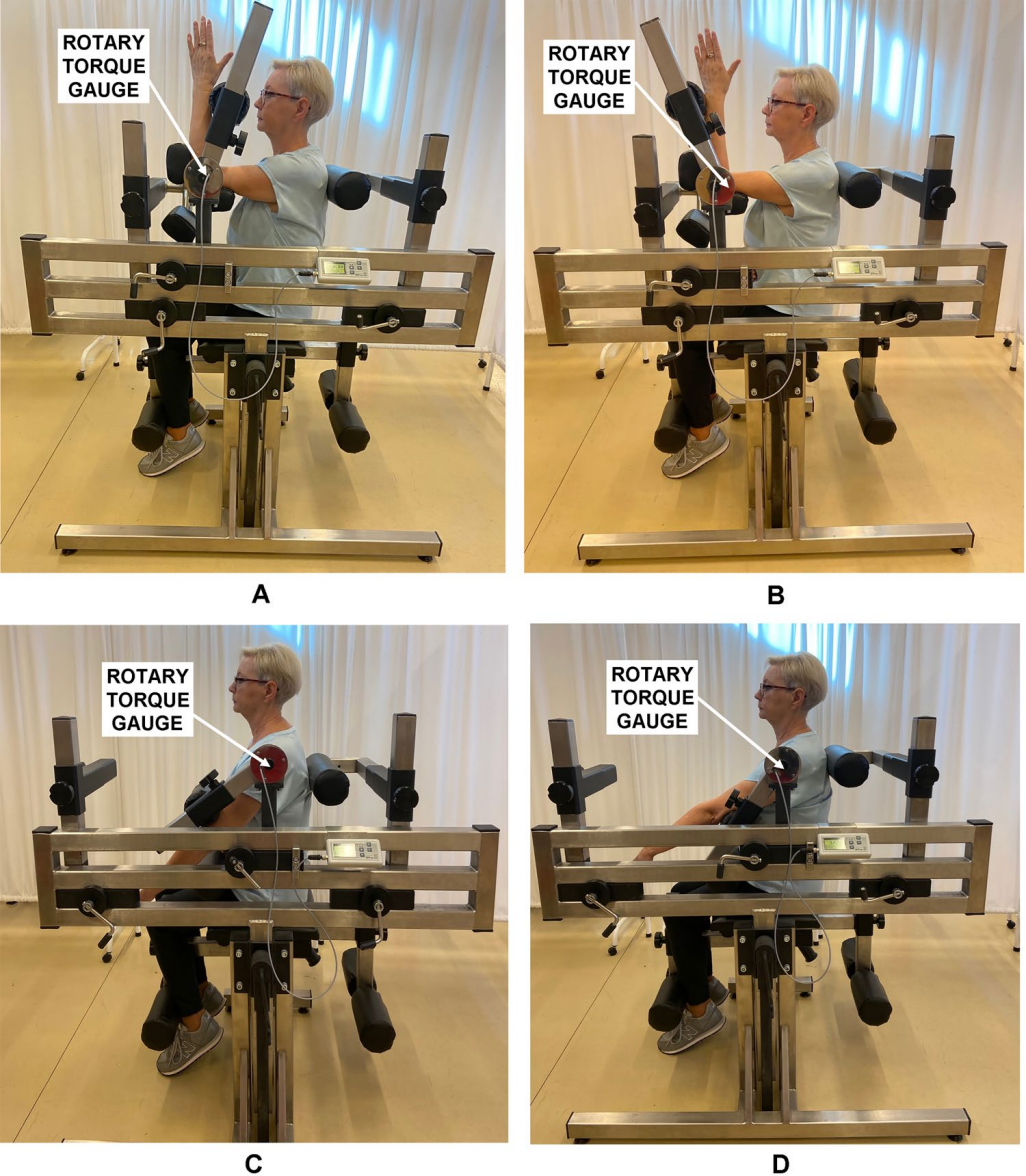
Torques of the elbow joint: flexors (**A**), extensors (**B**) and the shoulder joint: flexors (**C**) and extensors (**D**) measurement in a seated position.

Biomechanical assessments took place on one day, a few days before the training intervention and after their completion. The testing procedure used consisted of familiarization with the measuring equipment, followed by a trial test and then a main test. Torques of the flexors and extensors developed at the elbow and shoulder joint (Fig. 1) were measured in static conditions using an LR2-P tester from JBA Zb. Staniak. The bench is equipped with a torque meter with a measuring range of 500 Nm, with the maximum relative measurement error being less than 0.5%. The stand design ensures optimum stabilization of the torso, elbow and shoulder joints in a seated position (standard used in biomechanics laboratories). All measurements of torque at the elbow and shoulder joint were performed using the asymmetric technique, i.e. alternating measurements at the left and right joint. The maximum torque during the measurement was released in 1.5–3.0 s. The subject repeated each type of measurement 3 times with 30-s intervals, while the intervals between the four types of measurements were about 60 min. The highest of the three recorded torque values was selected as the final result of the measurement^20^.

The indicators in Table 2 were determined according to the following formulas:

1 $${\text{Flexion/extension }}\;{\text{ratio (\% ) }} = \frac{{{\text{Peak }}\;{\text{torque }}\;{\text{of}}\;{\text{ flexion}}}}{{{\text{Peak }}\;{\text{torque }}\;{\text{of }}\;{\text{extension}}}} \times {\text{100\% }}$$

2 $${\text{Index}}\;{\text{ of}}\;{\text{ torque}}\;{\text{ asymmetry (\% )}}\; = \;\frac{{{\text{|Peak }}\;{\text{torque}}_{{{\text{left}}}} \, - {\text{Peak }}\;{\text{torque}}_{{{\text{right}}}} |}}{{{\text{Maximum}}\left\{ {{\text{Peak }}\;{\text{torque}}_{{{\text{left}}}} {,}\;{\text{Peak}}\;{\text{ torque}}_{{{\text{right}}}} } \right\}}} \times {\text{100\% }}$$

### Statistical analysis

Data were collected as previously described in Wochna et al.^17^ and are presented as mean, standard deviation (SD), median and interquartile range. The normality of distributions was verified using the Shapiro–Wilk test and the homogeneity of variance using the Levene'a test. The t-test and Mann–Whitney U test were employed for normally and non-normally distributed variables, respectively, to evaluate the significance of differences between the groups of women. The t-test and Wilcoxon test, for normally and non-normally distributed variables, respectively, were used to examine the differences over the time (between the first and second times that subjects were tested). A 2 × 2 (group  ×  time) repeated-measures ANOVA was used to evaluate the influence of the pole type on the assessed indices in time. For interaction effects (“time × group”) and main effects (“time” and “group”), the eta-squared effect size was calculated. The effect size indicates the percent of variance explained by effects of the dependent variable. The Spearman’s rank and r-Pearson’s analysis for non-normally and normally distributed variables were used to calculate correlation coefficients. Interpretations of correlation coefficients for biomedical sciences were adopted^21^. Statistical significance was set at an alpha of 0.05 for all statistical procedures. The results obtained were analyzed statistically using Statistica v. 13.0 software (TIBCO Software Inc., Palo Alto, CA, USA).

### Ethics approval and consent to participate

The study protocol was approved by the Ethics Committee of Poznan University of Medical Sciences (No. 245/19). All participants gave written informed consent to participate in the study program.

## Results

With respect to age, there was only a tendency to significance of difference between the RSA and NW groups of women (mean ± SD: 68 ± 4.19 vs. 65 ± 3.40 years, respectively, percentage difference 4.4%, p = 0.051).

Table 1 presents the results of somatic characteristics and areal bone mineral density, and femur strength index, before and after the intervention in both groups of women. At the beginning of the study, there were no significant differences (p < 0.005) between RSA and NW groups with respect to somatic parameters, as well as to total fat, lean body mass and BMD measurements.

**Table 1:** Somatic features, areal bone mineral density and femur strength index before and after the intervention in the Nordic walking (NW) and resistance shock absorber (RSA) groups.

Parameters		Assessment at baseline	Assessment after the intervention
Body mass (kg)	NW	67.21 (10.39); 64.5 (61.10 to 71.00)	67.97 (10.45); 65.5 (60.9 to 71.60)*
	RSA	71.05 (7.92); 73.0 (66.00 to 77.60)	71.47 (8.29); 73.95 (66.60 to 78.50)
BMI (kg/m^2^)	NW	26.11 (3.11); 25.4 (24.0 to 27.70)	26.41 (3.09); 25.8 (24 to 28.30)
	RSA	27.35 (3.21); 28.55 (24.40 to 29.40)	28.48 (4.66); 29.25 (24.90 to 30.50)
Total fat (%)	NW	41.27 (4.15); 42.9 (37.40 to 43.70)	40.60 (4.16); 41.50 (37.90 to 43.20)
	RSA	41.02 (5.37); 40.40 (37.00 to 44.50)	40.40 (5.94); 40.35 (36.10 to 44.40)
Lean body mass (kg)	NW	38.19 (4.57); 38.07 (35.72 to 39.65)	38.50 (4.88); 38.78 (35.63 to 40.17)
	RSA	40.62 (3.14); 40.53 (38.35 to 42.69)	40.66 (3.49); 39.96 (38.91 to 42.61)
Total fat (kg)	NW	27.18 (6.28); 25.16 (22.65 to 32.55)	26.68 (6.43); 25.83 (21.81 to 30.62)
	RSA	28.73 (6.32); 30.45 (24.16 to 33.55)	28.11 (6.60); 29.66 (23.21 to 34.08)
Total BMD (g/cm^2^)	NW	1.08 (0.06); 1.10 (1.03 to 1.13)	1.08 (0.07); 1.11 (1.03 to 1.13)
	RSA	1.06 (0.09); 1.05 (0.99 to 1.15)	1.06 (0.09); 1.05 (1.00 to 1.15)
Forearm BMD (g/cm^2^)	NW	0.45 (0.05); 0.45 (0.43 to 0.48)	0.46 (0.06); 0.46 (0.41 to 0.51)
	RSA	0.42 (0.06); 0.43 (0.37 to 0.46)	0.43 (0.07); 0.45 (0.39 to 0.47)
BMD L_1_-L_4_ (g/cm^2^)	NW	1.08 (0.22); 1.04 (0.91 to 1.26)	1.07 (0.21); 1.03 (0.88 to 1.31)
	RSA	0.99 (0.14); 0.97 (0.89 to 1.03)	0.98 (0.15); 0.95 (0.85 to 1.05)
BMD total femur (g/cm^2^)	NW	0.86 (0.08); 0.83 (0.79 to 0.91)	0.86 (0.08); 0.83 (0.8 to 0.92)
	RSA	0.93 (0.15); 0.9 (0.86 to 1.1)	0.93 (0.16); 0.9 (0.82 to 1.1)
BMD femoral neck (g/cm^2^)	NW	0.81 (0.06); 0.81 (0.76 to 0.86)	0.82 (0.07); 0.82 (0.76 to 0.86)
	RSA	0.85 (0.13); 0.84 (0.75 to 0.97)	0.86 (0.12); 0.85 (0.75 to 0.95)
Femur strength index	NW	1.31 (0.27); 1.3 (1.1 to 1.5)	1.24 (0.23); 1.3 (1.1 to 1.4)
	RSA	1.45 (0.30); 1.5 (1.2 to 1.6)	1.55 (0.31); 1.5 (1.3 to 1.7)^#^
T-score L_1_–L_4_	NW	− 0.81 (1.82); − 1.1 (− 2.3 to 0.7)	− 0.91 (1.78); − 1.3 (− 2.5 to 1.1)
	RSA	− 1.55 (1.17); − 1.8 (− 2.4 to − 1.2)	− 1.68 (1.24); − 1.95 (− 2.7 to − 1.1)
T-score total femur	NW	− 1.15 (0.67); − 1.4 (− 1.7 to − 0.8)	− 1.16 (0.65); − 1.4 (− 1.7 to − 0.7)
	RSA	− 0.62 (1.22); − 0.8 (− 1.1 to 0.7)	− 0.65 (1.28); − 0.85 (− 1.5 to 0.8)
T-score femoral neck	NW	− 1.61 (0.47); − 1.6 (− 2 to − 1.3)	− 1.56 (0.49); − 1.5 (− 2 to − 1.3)
	RSA	− 1.36 (0.91); − 1.45 (− 2.1 to − 0.5)	− 1.28 (0.90); − 1.35 (− 2.1 to − 0.7)

Results are expressed as mean (SD); median (interquartile range); BMD, bone mineral density; * indicates a significantly different between terms of the study (p < 0.05);^#^ indicates a significantly different between RSA and NW groups (p < 0.05).

After the intervention, comparative analysis between both groups showed a significant difference with respect to the femur strength index (p = 0.008). Comparing the variables presented in Table 1 between the study terms, a significant increase was only found with respect to body mass in the NW group (p = 0.019). In the RSA group, no significant difference was noted between the terms of the study.

**Figure 2:**
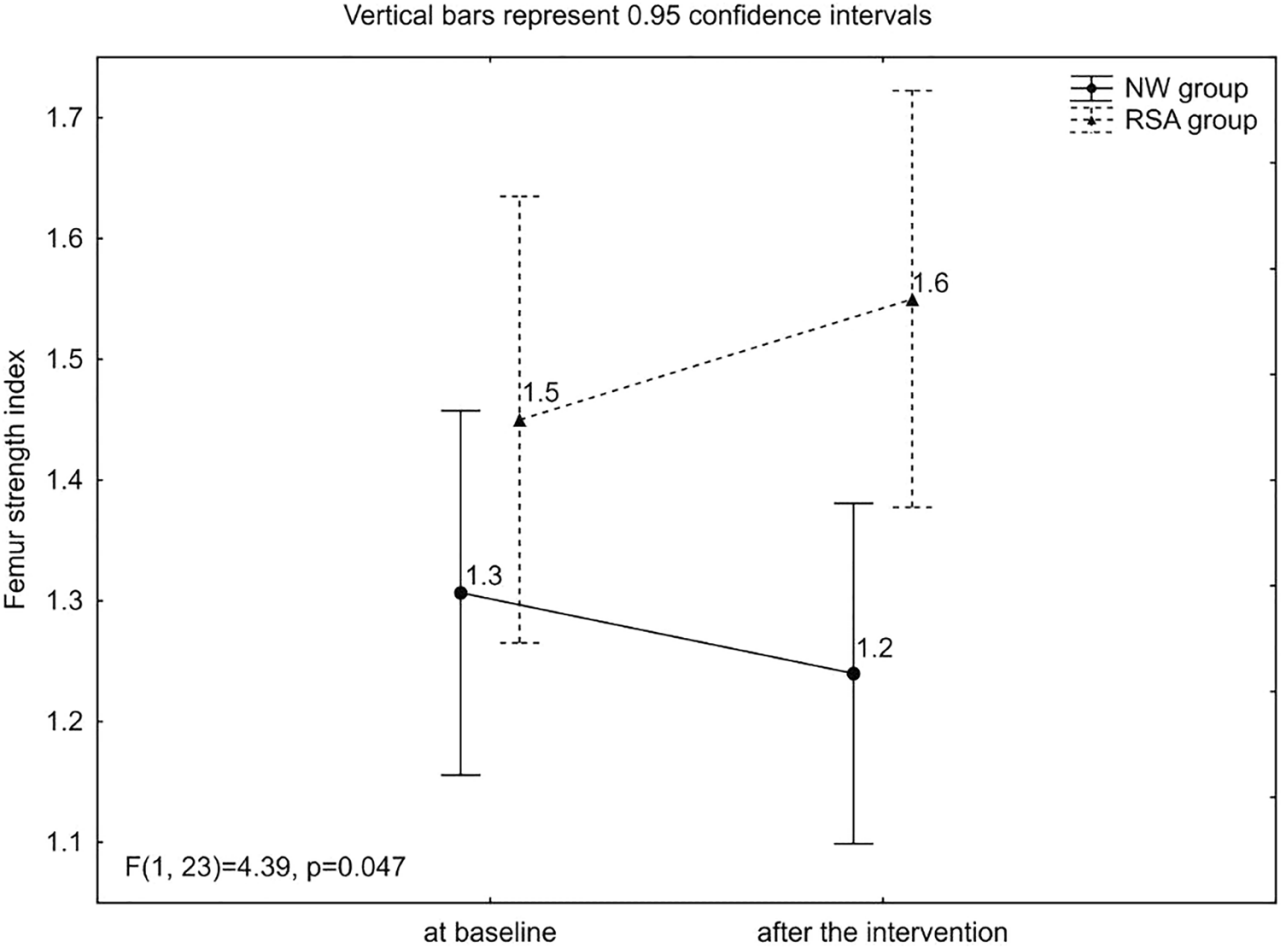
Mean values, confidence intervals and analysis of variance ANOVA for results of the femur strength index at baseline and after the intervention in groups of Nordic walking with classic poles (NW) and with poles with resistance shock absorber (RSA).

A 2 × 2 (“group” × “time”) repeated-measures ANOVA revealed a significant interaction effect with respect to the femur strength index at baseline and after the intervention between both groups, which was beneficial for the RSA group (F = 4.49; p = 0.047; ŋ^2^ = 0.16)—Fig. 2.

Table 2 presents biomechanical variables in both groups of women (NW and RSA) in two terms of the study. In the first term of the study, comparative analysis between the RSA and NW groups showed a significant difference with respect to the strength ratio of the flexors to extensors (1) in the shoulder joint of the left arm (p = 0.004). In the second term of the study, a significant difference between the RSA and NW groups was found only for the strength ratio but in the elbow joint of the left arm (p = 0.031).

**Table 2:** Biomechanical features before and after the intervention in the Nordic walking (NW) and resistance shock absorber (RSA) groups.

Parameters		Assessment at baseline	Assessment after the intervention
**Peak torque (Nm)**			
Elbow extension L	NW	17.2 (4.2); 17.2 (13.8–19.4)	19.1 (4.9); 17.5 (14.6–21.7)
Elbow extension R		19.0 (4.3); 19.4 (16.0–22.2)	20.5 (5.4); 20.8 (16.0–24.2)
Elbow flexion L		21.9 (6.0); 21.7 (17.5–24.5)	28.7 (6.8); 27.6 (23.4–34.9)**
Elbow flexion R		25.7(4.5); 25.9(21.9–29.0)	25.8(3.5); 25.9(23.1–28.4)
Elbow extension L	RSA	18.9 (9.2); 15.2 (13.0–21.1)	23.5 (7.3); 21.0 (19.8–23.1)*
Elbow extension R		19.5 (8.5); 16.2 (14.6–21.1)	22.8 (7.4); 23.4 (18.6–25.6)
Elbow flexion L		24.0 (8.2); 22.8 (18.6–29.6)	28.9 (7.1); 27.3 (25.6–31.2)*
Elbow flexion R		25.1 (9.8); 23.8 (17.7–29.6)	28.7 (5.4); 28.0 (25.6–30.7)
Shoulder extension L	NW	39.3 (6.1); 39.7 (34.6–42.2)	35.4 (7.8); 33.2 (30.7–42.2)
Shoulder extension R		36.4 (10.6); 38.3 (32.3–43.6)	37.4 (6.8); 36.9 (31.2–45.0)
Shoulder flexion L		27.5 (9.5); 25.6 (21.1–32.7)	28.5 (8.1); 29.0 (22.0–31.5)
Shoulder flexion R		35.0 (7.5); 36.0 (29.8–39.4)	39.6 (8.5); 38.8 (32.7–49.8)
Shoulder extension L	RSA	37.5 (13.7); 32.1 (27.1–42.5)	36.1 (11.1); 36.4 (30.1–38.3)
Shoulder extension R		36.3 (13.0); 30.7 (27.0–44.8)	36.3 (7.7); 35.2 (32.4–39.4)
Shoulder flexion L		35.0 (12.1); 32.5 (25.6–41.9)	27.1 (6.9); 28.0 (20.5–33.5)
Shoulder flexion R		35.6 (12.0); 31.3 (28.7–40.5)	43.4 (8.6); 43.8 (35.7–47.6)**
**Flexion/extension ratio (%)**			
Elbow L	NW	130.4 (29.8); 134.1 (111.9–159.1)	154.1 (32.1); 158.1 (141.0–170.1)*
Elbow R		137.8 (18.5); 132.0 (124.2–148.2)	133.9 (39.1); 116.4 (107.4–166.7)
Shoulder L		68.7 (15.6); 70.7 (57.2–77.5)	81.3 (16.9); 79.2 (67.0–96.7)*
Shoulder R		102.1 (29.0); 92.3 (83.7–114.9)	106.8 (19.0); 110.7 (93.1–116.9)
Elbow L	RSA	137.9 (49.6); 123.6 (104.1–156.2)	126.6 (24.0); 121.6 (113.3–134.1)
Elbow R		130.9 (20.9); 127.4 (115.2–151.5)	137.9 (52.5); 127.7 (104.3–143.5)
Shoulder L		96.7 (29.8); 90.0 (79.5–97.1)	77.9 (20.7); 74.2 (60.7–90.1)
Shoulder R		100.2 (14.5); 97.0 (90.4–106.7)	122.3 (24.6); 124.9 (98.3–141.7)*
**Index of torque asymmetry (%)**			
Elbow extension	NW	17.9 (12.8); 19.7 (5.4–32.5)	22.6 (12.0); 22.6 (12.2–34.3)
Elbow flexion		20.8 (11.0); 20.4 (15.2–31.7)	15.6 (11.7); 19.3 (4.1–25.8)
Shoulder extension		15.6 (15.3); 9.2 (5.6–18.9)	12.1 (11.5); 8.7 (3.2–17.2)
Shoulder flexion		23.4 (14.7); 22.8 (13.3–35.0)	29.0 (14.0); 35.5 (13.1–40.2)
Elbow extension	RSA	8.7 (7.5); 7.1 (3.8–9.5)	22.9 (18.3); 19.9 (7.0–22.7)**
Elbow flexion		20.4 (12.7); 20.3 (11.5–26.5)	12.5 (9.4); 12.2 (3.1–18.0)
Shoulder extension		15.8 (13.2); 13.4 (3.1–27.5)	19.5 (11.7); 19.1 (12.0–23.6)
Shoulder flexion		16.1 (12.4); 14.1 (8.9–24.9)	38.0 (7.6); 39.2 (29.7–41.8)**

Results are expressed as mean (SD); median (interquartile range); L—left, R—right; ** and * indicates a significantly different between the study terms (p ≤ 0.01 and p < 0.05, respectively).

When analyzing time’s impact on the variables (comparative analysis between both terms of the study) in RSA group, significant differences were found for the following biomechanical indices: the peak torque (PT) of flexors (p = 0.022) and extensors (p = 0.013) in the elbow joint of the left arm; the PT of flexors in the shoulder joint of the right arm (p = 0.007); the ratio of flexors to extensors (1) in the shoulder joint of right arm (p = 0.025); the index of torque asymmetry (2) of extensors in the elbow joint (p = 0.007) and flexors in the shoulder joint (p < 0.001). In the NW group, significant differences were found for: PT of flexors in the elbow joint of the left arm (p = 0.006) and the ratio of flexors to extensors (1) in the elbow (p = 0.045) and shoulder (p = 0.043) joints of the left arm.

**Figure 3:**
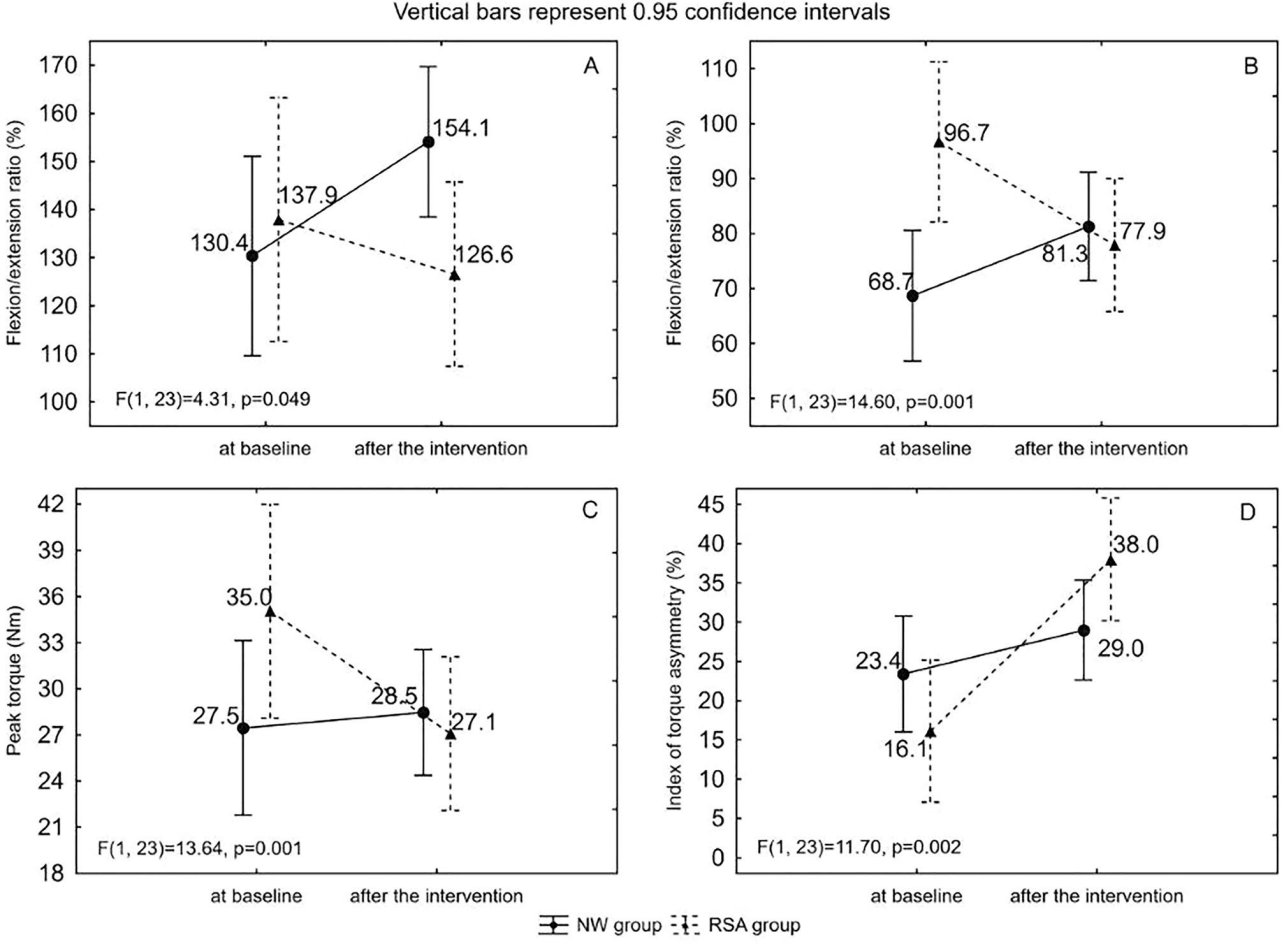
Mean values, confidence intervals and analysis of variance ANOVA for results of the strength ratio of flexors to extensors in elbow (**A**) and shoulder (**B**) joints, the peak torque of flexors in shoulder joint for left arm (**C**) and the index of torque asymmetry with respect to flexors in shoulder joint (**D**) at baseline and after the intervention in groups of Nordic walking with classic poles (NW) and with poles with resistance shock absorber (RSA).

A 2 × 2 (“group” × “time”) repeated-measures ANOVA revealed significant interaction effects (Fig. 3) with respect to the ratio of the flexors to extensors (1) in the elbow (F = 4.31; p = 0.049; ŋ^2^ = 0.16) and shoulder (F = 14.60; p = 0.001; ŋ^2^ = 0.39) joints of the left arm and PT of flexors in the shoulder joint (F = 13.64; p = 0.001; ŋ^2^ = 0.37) for the left arm. A significant difference was also found in the index of torque asymmetry (2) of flexors in the shoulder joint (F = 11.70; p = 0.002; ŋ^2^ = 0.34).

The correlation analysis of changes between pre-post intervention values of the femur strength index (Δ) with somatic features, or bone mass indices measured before the training program did not show any significant relationships in both the groups investigated (RSA and NW).

## Discussion

Our hypothesis that training with RSA poles, with additional resistance, will cause a significant response of the musculoskeletal system has been confirmed. We revealed a significant interaction between the groups of postmenopausal women and time of the intervention with respect to femur strength index with a beneficial effect in the RSA group and we observed the significant response of the biomechanical parameters. This result is important in terms of hip fracture prediction. In their study of 2506 women over the age of 50 years, Faulkner et al.^18^ found that femur strength index and hip axis length are significant predictors of hip fracture. In our previous study^17^, a significant improvement in femoral bone strength index was observed in postmenopausal women during 6 months of aqua fitness training. The duration of the presented study using the NW training program was relatively short (2 months), but the use of RSA poles was a sufficient load, for changes in the femoral strength index to occur.

There was found no significant influence of Nordic walking with RSA on bone mineral density values measured on the whole body, the femoral neck, forearm or lumbar spine regions. Several studies conducted mostly in unhealthy subjects show that NW is a beneficial type of physical activity, which positively stimulates bone metabolism, but may induce varied responses in the skeleton. In their meta-analysis, Rodrigues et al.^9^ included randomized controlled trials comparing walking to control individuals aged ≥ 50 years with low BMD and at risk of fractures. The effects on femoral neck BMD were not significant, although the authors did not provide a description of training session or exercise intensity, and they concluded that walking may need to be combined with other exercises for health benefits. Kelley and Kelley’s^22^ large meta-analysis showed that exercise does not improve femoral neck BMD in postmenopausal women. However, most of the studies that were included in their investigation used rather low impact types of activities, primarily walking, as an intervention.

Faulkner et al.^18^ suggested that measuring BMD alone may not provide the best predictive ability to detect the risk of hip fracture. The authors noted that the strength of the proximal femoral bone is a function of femoral BMD and also its structural geometric properties. In our study, the significant result of the training program in the RSA group with respect to the femur strength index may be explained by the use of poles, which caused a change of the body position during marching. As a result of acceleration, the body is tilted forwards with the center of gravity being shifted and a noticeable increase in the workings of the limbs and trunk. According to Marciniak et al.^13^ suggestions, we suppose that using RSA poles could generate great reaction forces on the skeleton^23^. The beneficial effects of walking with RSA training on the femur strength index appear to be important in the light of the fact that hip fracture injuries have been identified as one of the most serious health problems in older people^24^.

The present study examining the PT is important due to the fact that rapid force production is critical to improve performance and prevent injuries^25^. Our data revealed a significant interaction between the groups of postmenopausal women and the time of the intervention with respect to several biomechanical parameters. These results allow us to describe the locomotor model in RSA in relation to NW with classic poles because of some of their convergences. We suppose that changing the body position while walking with RSA poles compared to NW with classic poles may play a role in our results. Pellegrini et al.^10^ indicated that different NW techniques may influence the muscular and metabolic responses. RSA poles have a built-in shock absorber with a total length of 20 cm, therefore, the positioning of the upper limbs while marching with the RSA poles is different to that when using classic NW poles^15^. Moreover, pressing the shock absorber changes the length of the stick, which, when shortened to the maximum, reaches the length of a traditional NW stick. Therefore, the muscles must perform additional torque, overcoming the resistance of the elastic shock absorber in the pole. In our study, the increase in PT in the extensor of the elbow joint in the RSA group after the intervention may suggest that muscles were involved in overcoming resistance in this kind of poles. We observed that RSA poles are heavier than classic NW poles, and we suppose that it could generate extra work during upper limb flexion movement due to the weight of the poles. In their study conducted on adult women, Schiffer et al.^26^ indicated that increasing the weight of the pole alters the electrical activity of the upper limb muscles (biceps brachii, triceps brachii). The authors confirm the results of our article by stating that the use of heavier poles mainly leads to the increased involvement of the biceps brachii muscle. However, it should be emphasized that the triceps brachii muscle, which is responsible for straightening the limb at the elbow joint, must show less electrical activity compared to the flexor muscle, since the increased weight of the stick supports the extension of the upper limb. In our study, the increase in PT in the flexors of the right shoulder joint may be the effect of overcoming with weight of RSA poles. Surprisingly, a drop in the PT of the flexors in the left shoulder joint for the RSA group in interaction with the NW group was observed in our study**.**

We suppose that it could be the result of the load distribution on both the elbow and shoulder joints. However, this decline in flexor activity in the shoulder joint does not exceed the values for the similar age group provided by Danneskiold-Samsøe et al.^20^ in their study conducted on a large Danish population. Dalton and Nantel^27^ investigated a group of healthy older adults. Similar 8-week Nordic walking intervention resulted in various gait changes, including: a longer stride, faster gait, and increased power generation at the hip and power absorption at the knee. Consistent with this, Pellegrini et al.^28^ searched for mechanical energy patterns in Nordic walking. Their results showed that walking with poles might require isometric muscle coactivation in the upper body, as a part of the joint stabilization.

Furthermore, significant interactions in the ratio of the flexors to extensors (1) of the left elbow and shoulder joints were found between time and both groups (NW and RSA). Based on the average data published by Danneskiold-Samsøe et al.^20^ from their study of healthy subjects (60–69 years of age), we calculated that the ratio of flexors to extensors (1) in their population achieved a value up to 118.1% for the elbow joint and 63.8% for the shoulder. Our results exceed these values, in the first and also in the second term of the study, which indicate that the subjects’ joints were more loaded compared with the population examined by Danneskiold-Samsøe et al.^20^. Nevertheless, our study indicated that intervention with RSA poles caused a significant decrease in the ratio of flexors to extensors for the left arm (on average 11.3% and 18.8% for the elbow and shoulder joints, respectively), approaching the values described by the aforementioned authors. Within the NW group, we observed an increase in these parameters (by average 23.7% and 12.6% for the elbow and shoulder joints, respectively).

In the right shoulder joint, despite no significant interaction between RSA and NW with respect to the flexors to extensors ratio (1), the analysis showed a significant increase between the first and the second term of the study in the RSA group. We suppose that there is a load distribution between the left elbow joint and the right shoulder joint during intervention. Ultimately, the index of torque asymmetry (2) changed significantly with respect to the flexors in the shoulder joint in the group using RSA poles. This can most likely be explained by the phenomenon of limb domination and indicate dredging of asymmetry during the RSA training program. Furthermore, it may result in the occurrence of adverse asymmetry in spinal rotation movements, particularly in the lumbar^29^. In summary, biomechanical parameters suggest that walking using RSA poles caused a significant increase in asymmetry between left and right elbow extension and shoulder flexion. The asymmetry is related to higher torque values of the flexors in the left elbow and right shoulder joints after the intervention.

NW with RSA is an interesting form of activity that activates and improves musculoskeletal function^13^. However, caution is advised in recommending these exercises to the older adults. We suppose that this kind of activity may be too demanding effort for people with osteoarthritis. Therefore, embarking on exercises of this nature requires medical consultation.

The major limitation of this study is that it was carried out on a small sample. The authors did not expect that such a large number of people would ultimately be excluded from the study. Therefore, in future studies, the initial sample size should be increased. Extending the training program (up to e.g. 6 months) could contribute to a more objective assessment of bone mass response in DXA testing. Moreover, the additional techniques could be applied to verify changes in bone microarchitecture. In biomechanical aspects, in the future research, it could be interesting to focus more on ground reaction forces and the role of the dominant limb.

## Conclusions

The study showed that the NW with RSA poles led to beneficial changes in the femur strength index, which is important with respect to the prevention of fractures. NW with RSA may be an alternative to exercise with classic poles or to walking, especially for those who want to activate more their muscular system. However, the occurrence of asymmetry in biomechanical muscle parameters, which was observed using RSA poles, may suggest the necessity of systematic controlling the gait technique to avoid the adverse consequences of asymmetry in the rotation of the lumbar spine.

## Data Availability

The datasets used and analysed during the current study available from the corresponding author on reasonable request.

## References

[1] Yoo SZ, No MH, Heo JW, Park DH, Kang JH, Kim SH (2018). Role of exercise in age-related sarcopenia. J. Exerc. Rehabil..

[2] Martelli S, Beck B, Saxby D, Lloyd D, Pivonka P, Taylor M (2020). Modelling human locomotion to inform exercise prescription for osteoporosis. Curr. Osteoporos. Rep..

[3] Roy M, Grattard V, Dinet C, Soares AV, Decavel P, Sagawa YJ (2020). Nordic walking influence on biomechanical parameters: A systematic review. Eur. J. Phys. Rehabil. Med..

[4] Bullo V, Gobbo S, Vendramin B, Duregon F, Cugusi L, Di Blasio A (2018). Nordic Walking can be incorporated in the exercise prescription to increase aerobic capacity, strength and quality of life for elderly: A systematic review and meta-analysis. Rejuvenation Res..

[5] Kocur P, Wilk M (2006). Nordic Walking—a new form of exercise in rehabilitation. Med. Rehabil..

[6] Muollo V, Rossi AP, Milanese C, Masciocchi E, Taylor M, Zamboni M, Rosa R, Schena F, Pellegrini B (2019). The effects of exercise and diet program in overweight people—Nordic walking versus walking. Clin. Interv. Aging.

[7] Muollo V, Rossi AP, Milanese C, Zamboni M, Rosa R, Schena F, Pellegrini B (2021). Prolonged unsupervised Nordic walking and walking exercise following six months of supervision in adults with overweight and obesity: A randomised clinical trial. Nutr. Metab. Cardiovasc. Dis..

[8] Psurny M, Svoboda Z, Janura M, Kubonova E, Bizovska L, Lemos RIM (2018). The effects of nordic walking and slope of the ground on lower limb muscle activity. J. Strength Cond. Res..

[9] Rodrigues IB, Ponzano M, Butt DA, Bartley J, Bardai Z, Ashe MC (2021). The Effects of walking or Nordic walking in adults 50 years and older at elevated risk of fractures: A systematic review and meta-analysis. J. Aging Phys. Act..

[10] Pellegrini B, Bocci G, Zoppirolli C, Rosa R, Stella F, Bortolan L (2018). Muscular and metabolic responses to different Nordic walking techniques, when style matters. PLoS ONE.

[11] Park SK, Yang DJ, Kang YH, Kim JH, Uhm YH, Lee YS (2015). Effects of Nordic walking and walking on spatiotemporal gait parameters and ground reaction force. J. Phys. Ther. Sci..

[12] Kato T, Tomioka T, Yamashita T, Yamamoto H, Sugajima Y, Ohnishi N (2020). Nordic walking increases distal radius bone mineral content in young women. J. Sports Sci. Med..

[13] Marciniak K, Maciaszek J, Cyma-Wejchenig M, Szeklicki R, Maćkowiak Z, Sadowska D (2020). The effect of Nordic walking training with poles with an integrated resistance shock absorber on the functional fitness of women over the age of 60. Int. J. Environ. Res. Public Health.

[14] Lozano-Montoya I, Correa-Pérez A, Abraha I, Soiza RL, Cherubini A, O'Mahony D (2017). Nonpharmacological interventions to treat physical frailty and sarcopenia in older patients: A systematic overview—the SENATOR Project ONTOP Series. Clin. Interv. Aging..

[15] Marciniak K, Maciaszek J, Cyma-Wejchenig M, Szeklicki R, Stemplewski R (2021). The effect of Nordic walking training with poles with an integrated resistance shock absorber on the body balance of women over the age of 60. Healthcare.

[16] Domaszewska K, Koper M, Wochna K, Czerniak U, Marciniak K, Wilski M (2020). The effects of Nordic walking with poles with an integrated resistance shock absorber on cognitive abilities and cardiopulmonary efficiency in postmenopausal women. Front. Aging Neurosci..

[17] Wochna K, Nowak A, Huta-Osiecka A, Sobczak K, Kasprzak Z, Leszczyński P (2019). Bone mineral density and bone turnover markers in postmenopausal women subjected to an aqua fitness training program. Int. J. Environ. Res. Public Health.

[18] Faulkner KG, Wacker WK, Barden HS, Simonelli C, Burke PK, Ragi S (2006). Femur strength index predicts hip fracture independent of bone density and hip axis length. Osteoporos. Int..

[19] Yoshikawa T, Turner CH, Peacock M, Slemenda CW, Weaver CM, Teegarden D (1994). Geometric structure of the femoral neck measured using dualenergy X-ray absorptiometry. J. Bone Miner. Res..

[20] Danneskiold-Samsøe B, Bartels EM, Bülow PM, Lund H, Stockmarr A, Holm CC (2009). Isokinetic and isometric muscle strength in a healthy population with special reference to age and gender. Acta Physiol. (Oxf.).

[21] Yadav S (2018). Correlation analysis in biological studies. J. Pract. Cardiovasc. Sci..

[22] Kelley GA, Kelley KS (2006). Exercise and bone mineral density at the femoral neck in postmenopausal women: A meta-analysis of controlled clinical trials with individual patient data. Am. J. Obstet. Gynecol..

[23] Fukuchi CA, Fukuchi RK, Duarte M (2019). Effects of walking speed on gait biomechanics in healthy participants: A systematic review and meta-analysis. Syst. Rev..

[24] Marks R (2010). Hip fracture epidemiological trends, outcomes, and risk factors, 1970–2009. Int. J. Gen. Med..

[25] Morel B, Rouffet DM, Saboul D, Rota S, Clémençon M, Hautier CA (2015). Peak torque and rate of torque development influence on repeated maximal exercise performance: Contractile and neural contributions. PLoS ONE.

[26] Schiffer T, Knicker A, Montanarella M, Strüder HK (2011). Mechanical and physiological effects of varying pole weights during Nordic walking compared to walking. Eur. J. Appl. Physiol..

[27] Dalton C, Nantel J (2016). Nordic walking improves postural alignment and leads to a more normal gait pattern following weeks of training: A pilot study. J. Aging Phys. Act..

[28] Pellegrini B, Peyré-Tartaruga LA, Zoppirolli C, Bortolan L, Savoldelli A, Minetti AE (2017). Mechanical energy patterns in nordic walking: Comparisons with conventional walking. Gait. Posture.

[29] Ogurkowska M, Lewandowski J, Kawałek K (2016). Geometric parameters of the lumbosacral spine in elite rowers. Med. Sport.

